# Marsupialization of a Floor-of-Mouth Dermoid Cyst to Temporize Airway Distress

**DOI:** 10.7759/cureus.22781

**Published:** 2022-03-02

**Authors:** Dhruv Patel, Tyler J Ostrowski, Maria Faraz, Neil Gildener-Leapman

**Affiliations:** 1 Department of Otolaryngology, State University of New York Upstate Medical University, Syracuse, USA; 2 Department of Otolaryngology - Head and Neck Surgery, Albany Medical Center, Albany, USA; 3 Department of Pathology, Albany Medical Center, Albany, USA

**Keywords:** surgical management, airway emergency, airway mangement, dermoid cysts, head and neck infection

## Abstract

Dermoid cysts are benign masses of embryologic origin that can present in various anatomical locations throughout the human body. This article presents the case of a 30-year-old male who presented to our emergency department with complaints of tongue swelling accompanied by worsening dysphagia and dysphonia in the context of a chronic, midline mass in the floor of the mouth. Computed tomography (CT) imaging and surgical pathology of the mass ultimately revealed findings consistent with a dermoid cyst causing inferior displacement of the mylohyoid muscle. Initial management consisted of bedside drainage to temporize the airway, with marsupialization and in-office follow-up. Definitive treatment was achieved with surgical excision at a later date.

## Introduction

Dermoid cysts are benign embryologic masses that can present in a multitude of locations throughout the body. The pathologic origin of these cysts is debated; however, the most prevalent theory attributes these cysts to anomalies of early development. It is thought that during embryogenesis, entrapment of ectodermal tissue during embryonic fusion of the pharyngeal arches that give rise to head and neck structures leads to the formation of cysts. These cysts can be epidermoid, dermoid, or teratoid in nature. Histologically, dermoid cysts are lined by a keratinized stratified squamous epithelium and contain adnexal structures within their walls such as sebaceous glands and hair follicles [1]. Dermoid cysts can present anywhere in the body, with 7% occurring in the head and neck area and only 1.6% within the oral cavity [2]. These masses are usually asymptomatic; however, they have the potential to cause symptoms secondary to mass effect on surrounding structures. With the continued growth of a sublingual/oral cavity dermoid cyst, there is potential obstruction-related dysphagia, dysphonia, and ultimately dyspnea [3]. Generally, patients present with dermoid cysts in their second or third decade of life with a male to female ratio of 3:1 [4]. Here, we report the case of a dermoid cyst of the floor of the mouth that was managed with urgent drainage and marsupialization as well as subsequent routine surgical excision.

## Case presentation

**Figure 1:**
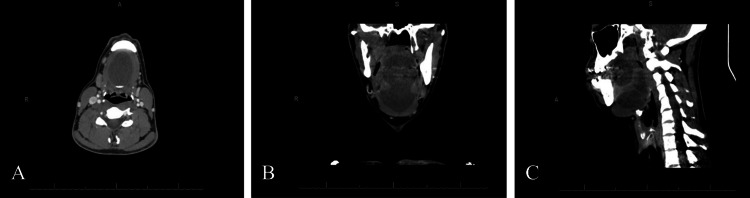
Contrast-enhanced CT neck-soft tissue with contrast of patient’s dermoid cyst at initial presentation to the emergency department. A: Axial view; B: Coronal view; C: Sagittal view

A 30-year-old male with a chronic floor-of-mouth mass was transferred to our emergency department from an outside hospital with a two-day history of acute oral swelling. He had known of the oral lesion since he was 15 years old and had avoided medical care since that time. At the first presentation, the patient denied any pain or difficulty breathing. However, he did endorse dysphonia and dysphagia, both of which prompted his seeking medical care. The patient was evaluated, and on examination, the midline mass significantly displaced the tongue posteriorly causing obstruction of the oropharynx and muffling of his voice. On arrival to the emergency department, the patient was afebrile with a mildly elevated white blood cell count at 10.1x 10^9^/L. The patient, who was noticeably nervous and sensitive to exam, refused flexible nasolaryngoscopy. Contrasted CT of the neck identified a well-defined hypointense 4.6 cm x 8.8 cm cystic structure with posterior wall calcifications in the submental space that did not invade the intrinsic tongue muscles but did cause inferior deflection of the mylohyoid muscle, most suspicious for a dermoid cyst (Figure 1). The radiodensity of the contents of the cyst was measured to be 32.4HU (SD 7.6HU; area of 7.8cm^2^) indicating fluid content. A bedside needle aspirate was performed of the cyst, which demonstrated 4+ non-enterococcus alpha hemolytic streptococci, 3+ presumptive coagulase negative streptococci, and 1+ *Rothia mucilanginosa *on wound culture. The patient was discharged on clindamycin 450mg three times daily for 10 days to treat the presumed infected dermoid cyst as he declined inpatient admission.

The patient returned to the emergency department two weeks later with worsened dysphagia, difficulty managing his secretions, and mild shortness of breath. At this point, an incision and drainage were performed in the emergency room with a small volume of debris released and some temporization of shortness of breath; 2mL of 1% lidocaine with epinephrine was injected into the right floor of mouth mucosa as a local anesthetic. An 18-gauge needle was used to aspirate approximately 20 mL of fluid after which an 11 blade was used to make a linear incision. The mucosal edges of this opening were marsupialized with a 3-0 chromic gut suture. The patient declined hospital admission and was not open to tracheostomy if needed for airway management. Three weeks after initial presentation, the patient was seen in the Head and Neck Surgery clinic. The floor-of-mouth incision site had closed, and the patient was again having difficulty tolerating his secretions and was mildly short of breath. At that point, the floor of his mouth was re-incised along the midline, 3cm in length, and copious caseous material was evacuated from the cyst until it was deflated and just the lining remained. The patient was immediately relieved, his speech improved, and he was able to breathe without restriction and tolerate his secretions. The cavity was packed daily with ½ inch plain gauze to keep the area of marsupialization open while surgery was scheduled in a routine manner.

**Figure 2:**
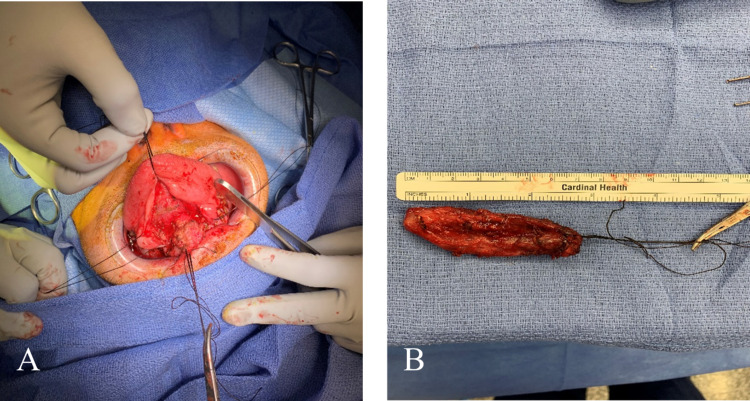
Intraoperative gross photographs of the cyst prior to excision (A) and immediately following definitive excision (B).

Excision of the dermoid cyst was performed three months after the initial presentation (Figure 2). The patient was nasally intubated, and surgery was carried out via a trans-oral approach. General anesthesia was induced. The patient was intubated transnasally. A cheek retractor was inserted into the oral cavity. A 2-0 silk suture was used to suture through the tongue at midline to retract the tongue superiorly. A monopolar cautery was used to cut the midline of the floor of the mouth avoiding injury to Wharton's duct. The incision was also extended anterolaterally about 1cm on each side. A 2-0 silk suture was used to suture on each side of the midline incision and retract the mucosa laterally. A combination of mosquito clamp, finger dissection, and Kitner was used to dissect around the cyst. The cyst was detached from the deep muscle of the tongue and the geniohyoid and sent to pathology. The deep musculature was closed with interrupted 3-0 Vicryl in layers to close dead space and then the mucosa was closed with 3-0 Vicryl in horizontal mattress. No drain was used. 

**Figure 3:**
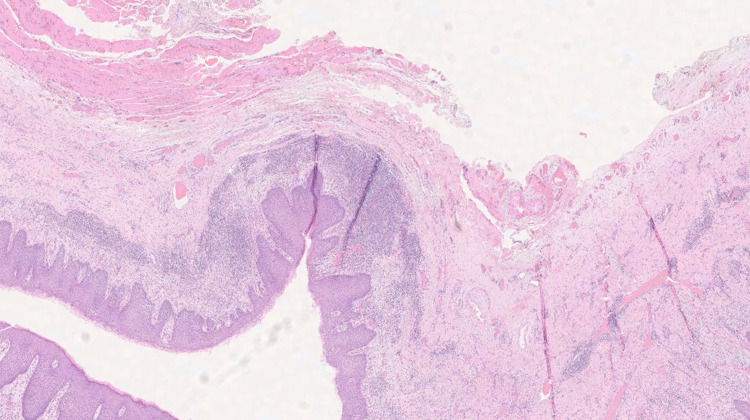
High-powered image of pathology slide of surgical specimen demonstrating a cystic structure lined by hyperplastic squamous mucosa containing focal adnexal structures with mural fibrosis, chronic inflammation, and focal intraepithelial acute inflammation.

Surgical pathology sections demonstrate a cyst lined by hyperplastic squamous mucosa. The wall of the cyst contained focal adnexal structures including sebaceous glands and scattered foci of smooth muscle fascicles with mural fibrosis, chronic inflammation, and focal intraepithelial acute inflammation. These findings are consistent with a benign oral dermoid autogenic cyst (Fig. 3). Adnexal structures, such as sebaceous glands, were also identified interspersed with scattered foci of smooth muscle fascicles.

The patient remained intubated overnight in the surgical ICU following the procedure due to significant tongue edema and was started on dexamethasone IV for 48 hours. The following day he was extubated without any complications. The patient showed no signs of dyspnea or dysphagia and was discharged home on postoperative day two. The patient followed up as an outpatient one week after the excision with minimal symptoms. Ultimately, the patient was lost to follow-up due to surrounding social issues. 

## Discussion

Dermoid cysts are uncommon congenital formations that can occur in diverse regions of the human body such as the head, neck, retroperitoneum, ovaries, and brain [5-9]. These cysts commonly present in the head and neck region; most of which, however, are diagnosed before the age of five years old [5]. The most prevalent theory for cyst formation postulates that they stem from entrapment of ectodermal-derived tissue during the fusion of embryonic structures along the midline symmetry axis [10]. In the case of head and neck dermoid cysts, entrapment can occur during fusion of the five pharyngeal arches and fascial processes that occur during weeks four to eight of development [1,10]. Dermoid cysts are comprised of keratinized stratified squamous epithelial lining, which contains adnexal structures such as hair follicles, sebaceous glands, and eccrine glands [7]. The presence of these adnexal structures is what differentiates a dermoid cyst from an epidermoid cyst. The third variant of these cysts, the teratoid cyst, develops from the entrapment of all three germ layers (endoderm, mesoderm, and ectoderm) and have the potential for malignant transformation [2].

Overtime, dermoid cysts can increase in size as secretions begin to accumulate. The symptoms associated with dermoid cysts of the sublingual oral cavity derive largely from the mass effect it imparts on the anatomy. With posterior dislocation of the tongue and obstruction of the oral cavity, symptoms of dysphagia, dysphonia, and dyspnea can develop. The large space-occupying effects can also impart restrictions in proper dental care. For the evaluation of a patient suspected to have a dermoid cyst, ultrasound and CT imaging provide valuable diagnostic and surgical planning information. Imaging coupled with fine needle aspiration can provide a greater confidence in pre-operative diagnosis [4]. Typical management of a dermoid cyst relies on surgical excision either via intraoral approach or a combined intraoral and transcutaneous approach. Although there is no set standard in regard to surgical approach, it is generally determined by cyst location (infra- or supra-geniohyoid) and size. Dermoid cysts that are inferior to the geniohyoid and/or large are surgically excised via intraoral and submental access [4]. This case report is notable for the acute bedside management that was undertaken to temporize a progressing airway obstruction prior to definitive surgical removal. The procedure described allowed for the evacuation of the dermoid cyst contents, reduction in mass effect, initial biopsy analysis, and time to further plan for a definitive surgical excision.

## Conclusions

Dermoid cysts are benign masses pathologically characterized by keratinized stratified squamous epithelial lining that contain structures derived from several germ layers. When these cysts occur in the head and neck 3 of 4 region, they can cause multiple health issues including dysphonia, dysphagia, and dyspnea even the absence of invasion into local structures. The case presented in this report involves the identification and more so the unique treatment of a dermoid cyst including acute bedside prevention of a worsening airway obstruction in conjunction with mass analysis for evaluation and planning of definitive surgical excision.
